# Talking about depression during interactions with GPs: a qualitative study exploring older people’s accounts of their depression narratives

**DOI:** 10.1186/s12875-018-0857-8

**Published:** 2018-11-03

**Authors:** Isabel Gordon, Jonathan Ling, Louise Robinson, Catherine Hayes, Ann Crosland

**Affiliations:** 1 Faculty of Health Sciences and Wellbeing, University of Sunderland, City Campus Chester Road, Sunderland, SR1 3SD UK; 2 Newcastle University Institute for Ageing and Institute for Health & Society, Newcastle University, Newcastle upon Tyne, NE4 5PL England

**Keywords:** Primary care, Older people, Depression, Narratives, Communication, Qualitative

## Abstract

**Background:**

Older people can struggle with revealing their depression to GPs and verbalising preferences regarding its management. This contributes to problems for GPs in both detecting and managing depression in primary care. The aim of this study was to explore older people’s accounts of how they talk about depression and possible symptoms to improve communication about depression when seeing GPs.

**Methods:**

Adopting a qualitative Interpretivist methodological approach, semi-structured interviews were conducted by IG based on the principles of grounded theory and situational analysis. GPs working in north east England recruited patients aged over 65 with depression. Data analysis was carried out with a process of constant comparison, and categories were developed via open and axial coding and situational maps. There were three levels of analysis; the first developed open codes which informed the second level of analysis where the typology was developed from axial codes. The typology derived from second level analysis only is presented here as older people’s views are rarely reported in isolation.

**Results:**

From the sixteen interviews with older people, it was evident that there were differences in how they understood and accepted their depression and that this influenced what they shared or withheld in their narratives. A typology showing three categories of older people was identified: those who appeared to talk about their depression freely yet struggled to accept aspects of it (Superficial Accepter), those who consolidated their ideas about depression aloud (Striving to Understand) and those who shared minimal detail about their depression and viewed it as part of them rather than a treatable condition (Unable to Articulate). The central finding was that older people’s acceptance and understanding of their depression guided their depression narratives.

**Conclusions:**

This study identified differences between older people in ways they understand, accept and share their depression. Recognising that their depression narratives can change and listening for patterns in what older people share or withhold may help GPs in facilitating communication to better understand the patient when they need to implement alternative approaches to patient management.

**Electronic supplementary material:**

The online version of this article (10.1186/s12875-018-0857-8) contains supplementary material, which is available to authorized users.

## Background

In the UK between 4.6 and 9.3% of older adults experience major depression, and an average of 17.1% experience depressive disorders [1]. Of these fewer than one in six will talk to GPs about their symptoms and only half will receive suitable treatment [2] due to poor detection and older people’s reluctance to seek help due to isolation or a “nihilistic” attitude [3]. In the UK, primary care is the first point of contact for many older people with health problems, with 22% visiting their GP in a two-week period [4]. Evidence goes some way to explaining why older people can be reluctant to talk to GPs and accept treatment for depression [5–7] yet barriers remain between older people and GPs where the acknowledgement and prioritisation of depression as a legitimate problem requiring medical support prevail [8–14].

Even though older people respond well to probing about their mood by primary care physicians [15] they struggle to reveal depression to GPs [9, 16] or to verbalise their views and preferences relating to the management of their depression [17, 18]. This reluctance conflicts with the value placed on older people talking to GPs about depression whether it is expressing it in terms of situational factors such as loneliness and isolation [12, 13] negotiating how it could be framed as an acceptable concept [11] or establishing justifications for their low mood [10].

Older people’s perceptions and beliefs about depression can influence ways they communicate to GPs [9, 10, 13] and they can be unwilling to talk about depression especially when it is normalised in comparison to other health problems [11]. While some older people value talking about depression as a form of help [19] for many it carries a stigma which ultimately is a barrier to seeking help from GPs [9, 10]. Perceptions of depression being a personal responsibility to overcome independently may also deter patients in asking GPs for help, especially when they need help with other physical or social problems [10].

Conceptually older people may not recognize depression as an illness needing treatment [20], rather seeing it as due to circumstances that often accompany old age, such as loneliness [12], and therefore a non-medical problem which is not a GP’s responsibility [6, 21] or a moral failing [14].

The use of language and the way GPs and patients talk about depression, in the context of GP practice settings in primary care, has been highlighted as particularly important with older people [8, 13, 15]. For example, those with chronic conditions are likely to frame their depression in the context of their life stories and may describe depression in terms of situations and experiences [10] or loneliness [12]. However, describing depression as a chronic, physical disease which older people find acceptable and normal in later life rather than a psychiatric brain disease is likely to facilitate communication with GPs [8, 22]. They may also see physical illness as a priority over their depression and that a GPs’ capacity to help is limited unless they are suicidal [10] which would deter them from raising depression with GPs. These contrasting rationales for help-seeking indicate complexities and differences underlying how older people validate depression and the impact this has on what they tell GPs.

Evidence shows how depression in later life can slip through the net, where the medical framework used for its detection does not always fit with ways older people frame and talk about it [6, 8, 12, 14, 23] including the complexities of them asking for help with depression in the context of other health or life problems [5, 9–11, 13]. There is little suggestion in the evidence of how GPs can accommodate these factors when older people with depression talk to them. More understanding of the differences and complexities in ways older people conceptualise depression and the impact this has on ways they talk about it would assist GPs in detecting depression according to how older people frame it. The aim of this study was therefore to obtain older people’s accounts of how they talk about depression or possible symptoms of depression in order to help improve communication between older patients who have depression and their GPs. Particular focus was on differences in their depression narratives and the factors that influence these.

## Methods

### Ethical considerations

Ethical approval was obtained from the local NHS National Research Ethics Service (NRES) and from Sunderland and North Tyneside CCGs where practices that recruited participants were located. Approval was also obtained from the University of Sunderland Research Ethics Committee. IG obtained an enhanced disclosure Criminal Records Bureau (CRB) check due to working with a vulnerable group.

### Study design

A qualitative study was conducted as part of a PhD. A grounded approach was used; this was informed by the more recent work of Adele Clarke [24] rather than traditional versions by those including Glaser and Strauss [25] that require the researcher to exclude preconceived ideas. Clarke’s methods seek to uncover multiplicity, the fluidity of ideas and points of difference rather than commonality; here they were used to facilitate recognition of complex, changing experiences of depression in later life and the range of differences that older people report in their experiences of having depression. Empirical data were generated through in depth semi-structured interviews [26] based on a topic guide developed from the literature. Observational notes were made by IG during interviews to support analysis. No relationships were established with participants prior to the study commencing.

### Recruitment and sampling

Letters inviting GP practices to recruit patients were sent via NyReN (Northen and Yorkshire Research Network) to 169 practices along with information about the study explaining reasons for the research and what would happen. GPs working in three practices agreed to recruit patients; at their discretion they identified and sent invitation letters and information to patients over 65 with depression. Data collection, analysis and sampling were carried out iteratively by the lead author.

The first five respondents were interviewed, thereafter a theoretical sample of older people were selected using demographic data which indicated their potential to help explore further or discount ideas being developed. Early interviews (P1–5) indicated a need to gather data from participants who varied in age, gender and socioeconomic status. Accordingly, participants were sought for the second set of interviews (P6–10), which in turn indicated a need for data from those living in deprived inner-city areas and/or who had experienced long term or severe depression. Participants were sought for the third set of interviews as such (P11–16). Selecting patients on the basis of their age and severity/duration of depression was at the discretion of GPs and disclosed by participants at the time of interview if they were willing. The sample size was determined by the number of participants needed to achieve saturation and allow for the production of a full and detailed account of the data.

### Interview schedule

The initial topic guide [see Additional file 1] included open ended discussion points and prompts to encourage participants to disclose as much information as they felt comfortable with. This was developed as interviews and analysis progressed, becoming increasingly focused on participants’ depression narratives and influences on this, the impact of other people including GPs on their depression narratives, what the differences in their views of depression and its management were, content of their narratives and when and how their views changed, their personal experiences of depression, views of depression and how they felt about seeing GPs for depression. The grounded approach ensured care was taken to use the same vocabulary as the older people as prompts during the interview. After early interviews and analysis (P1–5), a further set of participants were recruited (P6–10) to inform proceeding interviews. This process was repeated with a third set of participants (P11–16) until data saturation. Written consent was obtained prior to data collection and interviews were transcribed verbatim by IG and an independent transcriber.

**Fig. 1:**
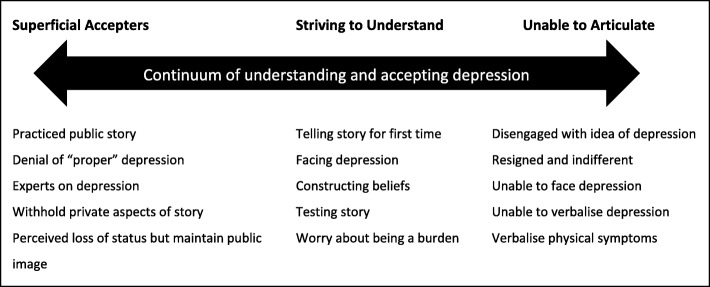
Typology of older people showing different positions of understanding and accepting depression on a continuum. Description: Visual representation of the typology of older people, showing three categories of older people and their positions on a continuum. Key characteristics of each category are listed underneath

### Analysis

Transcripts were initially coded and analysed iteratively by IG using the constant comparison method and stepped model of grounded theory [25] where three levels of analysis were undertaken. The constant comparison method informed theoretical sampling of participants where early ideas were tested with subsequent participants and found to either support or disconfirm developing ideas. Coding and interpretations were inspected and discussed by three others in the research team at monthly meetings and for each level of analysis in a process of triangulation [27, 28] to increase validity. Interpretations were available to participants on request but this was not taken up and no feedback was given.

In line with the stepped model of grounded theory three levels of analysis were carried out. Open codes were derived from the data during first level analysis and emergent axial codes noted, grouped together and categorised. This informed the second level of analysis where possible themes were explored further with later participants. These themes were supported with more data were developed into axial codes where a typology of older people with characteristics found in their narratives formed three distinct groups positioned on a continuum.

In the larger PhD study typologies for both older people and GP were developed. Third level analysis brought these two data sets together in a theoretical model [29]. The typology developed from second level analysis of older people’s data only is reported here as their views are underrepresented and seldom reported in isolation. Doing this underlines the importance of their voice being heard and allows an in-depth account of ways they communicate about depression.

A series of situational, social and positional maps [24] were used to explore the contextual, background and key influences in the data and enrich interpretations. Observations made during interviews (IG) were also considered in analysis and assisted the development of groups in the typology.

### Findings

Interviews were completed with 16 older people at their homes and lasted 1–2 hours; only participants and the researcher were present in all interviews. The sample consisted of 10 women and 6 men with a reported age range of 67–88 years. Data from all participants was included in the analysis plus observational notes made by IG during interviews; no participants refused to participate or dropped out, and there were no repeat interviews. No characteristics about the interviewer (IG) or influences over participants’ views were reported. Described here is a summary of axial codes derived from first level analysis of interview data: components of narratives, constructions and experiences of depression, narrative agendas. This leads to the typology consisting of Superficial Accepters, Striving to Understand and Unable to Articulate categories.

### Components

All participants recognised their narratives of depression were made up of different components which they shared or withheld from others. The most commonly cited components derived from open codes identified in early analysis of data were: descriptions of what depression feels like, what depression means, explanations for its cause, minimising depression, private and public stories and preferences in what to tell GPs. Differences between the ways older people talked about depression, language they used, what they told and withheld were prominent and began to define categories of the typology.

### Constructions and experiences

While all participants described their constructions and experiences of depression they did so to differing degrees of detail using a variety of vocabulary. Not all used the term depression saying it was an inaccurate reflection of the problems they were experiencing and offered their own explanations for it. Some did not see depression as a medical problem or minimised theirs compared to others’ who they saw as having “proper” (P3) depression. Others’ ideas about their depression did not appear as concrete where they were starting to put their experiences into words or questioned what had happened to them rather than providing explanations or views.

### Narrative agendas

Participants appeared to have differing agendas in their narratives of depression; some focused on certain aspects of their story regardless of any interview prompts, others were less focused and seemed to talk through ideas without any obvious direction. The remaining participants’ narratives were brief and closed, with conversation about depression minimal. All explained their reasons for telling their story in certain ways; they were motivated by how others saw them, telling their story for the first time, consolidating their ideas or regaining control in some way. Some did not want to talk directly about depression yet their narratives about their life contexts revealed how they saw depression in themselves and the impact it had on what they shared and withheld about depression.

*“I want to forget [experiences of depression], I don’t want to think about it cos it just brings it all back to me.”* (P1)*.*

Further data analysis led to the identification of a typology consisting of three distinct categories: Superficial Accepters (*n* = 5), Striving to Understand (*n* = 6) and Unable to Articulate (*n* = 8). These categories were shaped by the way older people spoke about depression in their interviews, the way they reported telling their stories of depression to other people including GPs, information about their depression they shared and withheld in comparison to each other and what they said about their experiences of seeing GPs for depression. Geographical location, affluence, reported severity/duration of depression and participants’ ages were not found to be influential over the categories.

The typology indicates variation in participants’ understanding and acceptance of depression and in their constructions and narratives at different positions on a continuum (Fig. 1). The continuum is used to emphasise that older people’s positions are not fixed, and that flexible, “porous” [24] boundaries are likely to exist between categories. This flexibility accounts for participants who describe change in ways they conceptualise their depression during their life prompting a move between categories, or where participants display characteristics in their storytelling from two categories.

*“all of a sudden the ECT [electroconvulsive therapy] had obviously sorted out my head and I said to myself, ‘look, this is silly, they are obviously not treating you for cancer they are treating you for mental problems’. Once I realised that then it [the depression] started to sort itself out.”* (P8)*.*

Analytical maps were used to set out multi factorial relationships identified between open and axial codes, around which analytical memos were developed to form categories of the typology. These were based around components of their stories they shared and withheld, their constructions and experiences of depression, their narrative agendas and perceived indications of change in ways they talked about depression.

### Superficial accepters

All participants in this category talked of their depression willingly and it appeared they had accepted it. Further probing revealed denial of having depression where they either compared themselves to others with depression in its “true form” (P5), stated theirs was not a “proper” (P3) illness or commented on others with depression appearing not to see themselves in the same category.

*“I would feel a bit down but not to the state that I couldn’t get out of the chair like I’d seen in my wife.”* (P5).

*“I never called it as depression I just felt awful, you know, you feel sad, I think sad is the word that I’ve always used.”* (P15)*.*

This tendency to deny or minimize their depression conflicted with the open and accepting way they initially talked about it. With probing, some comments revealed insecurities and stigma about having depression, which possibly stemmed from concern about a negative effect on their outward image.

*“People don’t want to listen to you, or worry about your illness, that’s how I feel.”* (P4)*.*

Many of these participants also revealed a stoical desire to hide their depression from their community or workplace for fear it would influence others’ perceptions of them, also indicating they may accept their depression only partially.

*“I still did my job alright you know, I didn’t have any problems. If I did I didn’t let anybody know about them I can tell you… It was a secret. My secret you know, I just got on with my job. Was never off sick.”* (P14)*.*

They demonstrated an inflexible agenda in their interviews, returning to the same topics regardless of any probing. For example, they gave detailed explanations about why they had depression, ensuring their reasoning and convictions about their depression were understood. This component of their narratives appeared practiced and lacked detail of their feelings or their views, as if they had prepared it for other people.

*“I would like to know what’s causing it… I have been very successful - I have been a head in four schools, successful as an artist, if I say so myself I am well liked in the village… so there is none of those things. It’s just…tiredness.”* (P9).

Many in this category portrayed themselves as experts on depression by reporting they had greater knowledge of their depression than healthcare staff and/or that their symptoms were not fully understood. In doing this some revealed anger and distrust of healthcare staff, expressing dissatisfaction with many aspects of their treatment or with doctors for putting a label on their depression.

*“Have you got any ailment that you always think oh well I know more about that than any of the doctors do? Well it’s the same thing …. I’ve given a lot of thought to it and read [about] it myself and understand exactly what my condition is, but a lot of them don’t.”* (P3).

Participants frequently referenced achievements in their education or careers. They often described facing a loss of status in some way, through career or position in family, and seemed to attach a lot of importance on how they presented themselves to others. Their style of talking about their experiences of depression focused on presenting facts, without giving much detail of how it felt or their views of it; they also tended to be articulate and the public components of their depression narratives were spoken with conviction. Although participants in this category were mostly men this did not appear to influence other characteristics; their willingness to talk about their experiences may simply have been because they were more sociable or better socialised through having a strong support network (as described by women) or a prominent career (as described by men).

### Striving to understand

A key characteristic of this category was that many used the interview to talk about their depression for the first time to anybody in such terms, or in any depth outside GP consultations. Some reported their motivation for taking part to be to offload to somebody or to “come clean” (P13). These older people were the most emotionally open of all categories in the typology when describing their thoughts and showing their feelings about depression.

*“I’ve talked to you more than I’ve ever talked to anybody [about my depression].”* (P15).

Some participants described testing different explanations for their depression aloud or practicing their story, sometimes as a way of reconnecting with other people. In doing this they appeared to be coming to terms with what had happened to them and were trying to clarify and articulate their understanding of depression.

*“I was detached from everybody else, I didn’t know why, and I didn’t know how then to reconnect and re communicate, it was very difficult.”* (P10).

Participants’ narratives were often long with little need for prompting; they tended to start talking immediately on contact, seemingly determined to tell their story. Their narratives could sound confused when they were remembering chronological events or what had happened to them, and they often appeared lost in their thoughts during interviews.

All participants in this category explored possibilities around their constructions of depression aloud. Though this category’s constructions of depression were not as developed as others, they described seeing it as an illness, a weakness of character, a normal feeling in old age and even questioned whether depression existed as a concept at all. They tended to alternate between the labels for depression possibly as they were still establishing their ideas and may not have been certain why they had been diagnosed with depression. Their uncertainty led to numerous contradictions in their narratives, but also prompted changes in their perspectives influenced by consolidating their ideas in the interview. For example, one participant had seldom spoken to anyone about her depression “*I’ve talked to you more than I’ve ever talked to anybody”* (P15) but by the end of the interview she felt older people with depression should *“talk to their family if they can and get them to understand; a lot of people don’t understand depression.”* (P15). Similarly, another interviewee described how he told his GP about his depression initially by talking about his physical symptoms,

*“I didn’t tell him [the GP] the details I just said, it started off with me feet and then I got a rash up me back and even in my face.”* (P13).

By the end of the interview P13 was calling his condition depression and seemed more comfortable talking about it in terms of emotional experiences.

People in this category were clear that the right timing was essential to opening up about their depression. Many described their interviews as a starting point to talk to others about their depression despite uncertainty about being ready or concern about being “a burden” (P13). Others spoke of having to “face up” (P8) to their depression or having ignored it in the past now felt ready to explore it further. Bereaved participants were common in this category, reporting this as a trigger for their depression but needing to pass through a crisis point before they could look back and reflect on their experiences out loud and share this with someone else.

*“She [the counsellor] said what I want you to do is write a letter, put your thoughts on paper, and, you know, I couldn’t do that, not at that time I couldn’t, I was too upset, like.” (P6).*

*“I intend to come clean today, because I tend when the family ring me up I’m always alright, even when I’m not, and…I don’t want to be a burden.”* (P13).

This category may not have established what they felt comfortable sharing and so switched between private and public components of their narratives. For participants in this category the opportunity to establish what was private and what they were willing to share in hindsight of depression appeared empowering as their depression narratives grew increasingly confident during interviews. This suggested that confirming their story aloud may have helped regain a sense of control which they typically described having lost when depressed.

*“If I needed to ask them for their support they would be absolutely furious that I hadn’t done it [earlier], but my feeling was, I’ve got to cope with it… It’s just silly, but if I told my son that he would have said mum, for heaven’s sake…”* (P10).

### Unable to articulate

Older people in the Unable to Articulate category had difficulty articulating their experiences, understandings or feelings about having depression into words, and appeared disengaged with the idea of it. They accepted they had depression and appeared resigned to it. This category revealed little more than the basic facts about their depression and while they cited a number of traumatic life events, they provided minimal detail of these by closing conversations. They described dealing with depression by blocking out trauma or disconnecting from it, which led them to withhold much of their depression narratives.

*“I want to forget, I don’t want to think about it cos it just brings it all back to me.” (P1).*

*“My family history is so appalling…it’s important that you know. I probably had one of the unhappiest childhoods [I’ve] ever heard of in my life… father committed suicide, sister committed suicide, mother who attempted suicide on numerous occasions… But that childhood, whether that has any bearing, I don’t know.”* (P9).

Willingness to talk was low among participants in the category but there was variation. All tended to open up more about their daily lives or activities and doing so gave some insight into their constructions of depression, underlining the importance of life narratives for this category. It was common for participants to start talking about an experience of depression then quickly draw the story to a close.

*“I sat here for weeks you know, couldn’t go out anywhere it affected me so much, but luckily I’ve got over it.”* (P2).

Two participants avoided talking about depression completely in interviews but still gave insight into their depression. P12 focused on heartbreak from losing people she loved during her life and this was how she defined her depression. Her narrative was stoical and she came across as detached from her depression, not wanting to talk about it at all. Similarly, P1 avoided talking about her depression, instead talking about pain in her back which seemed to be a way of expressing her feelings about her depression. The most extreme example within this category was P11 who said very little, and then only saying a few words about her life with heavy prompting. She appeared numb to her feelings and not to care about her depression or herself any more. She listed traumatic events that had happened during her life with brief, closed statements and minimal explanation.

There was a feeling within this category that their depression had completely taken them over for a long time, that they had no hope of things changing and no desire for exploring their own feelings and understanding depression. They described other people (e.g. doctors or family) or the medication to be in control of most aspects of their lives, and when asked how they felt about having depression said.

*“Well I’ve more or less accepted it, I’ve not got much choice.”* (P11).

People in this category had relatively fixed views on the reason for their depression (e.g. their personality) indicating they had long accepted the idea of having depression but were less likely to describe pivotal events or decisions to change their narratives than other categories.

They were unlikely to use the label depression, instead not talking about it directly or expressing it through physical symptoms such as *“heartbreak”* (P12) or *“my back”* (P1). P16 described many physical problems including tinnitus and pain on her face, explaining her depression by talking about these; she did not seem able to face her psychological distress or explain it any other way.

Participants in this category reported severe episodes of depression throughout their lifetimes managed in both primary and secondary care. They all described trying a range of medications and treatments appearing resigned and disconnected from these experiences. Their views were passive about how their depression was managed and their descriptions suggested they were used to doctors making decisions without their involvement.

## Discussion

### Summary of findings

Our findings highlight clear differences in the ways in which older people talk about their depression and provides a typology categorising their approach according to their narratives and how their understanding and acceptance of depression underpins this. This underlines a need for flexibility in the help provided to older people with depression and may provide opportunities for health care professionals to improve communication and understanding of how older people make sense of their depression.

Focus on older people’s depression narratives addresses an area known to be a problematic between older people and GPs [9–11, 13]. Older people value talking through their depression over biomedical treatments which may be less acceptable to them [5, 9, 19]. This study offers a framework to support GPs in understanding the complexities of the narratives older people may bring to consultations which tend to focus on their life story [10]. The typology here could also be considered for use outside the GP consultation by non-clinical staff, a factor on which importance is placed elsewhere [9].

This study supports literature suggesting that older people communicate their symptoms in line with their perceptions of depression [6, 23] and that this can deter them from asking for help [8, 13, 19]. It builds on evidence showing a need for patients to gain an understanding of their depression including its cause and what has happened to them [30, 31] so they can explain their experiences to others [32]. Here, older people’s acceptance of depression was a key influence for what they told GPs, where varying degrees of acceptance were a reason to tell or withhold parts of their story. Some struggled to recognise depression in themselves, typically denying or attributing it to something else (Superficial Acceptors), others were open to accepting it but needed to make sense of what had happened to them first (Striving to Understand) whereas those who had accepted it appeared to have reached an impasse and saw no way of progressing (Unable to Articulate). The resulting patterns of storytelling may indicate a way for GPs to open a dialogue with older people about help they would find acceptable rather than relying on them reporting symptoms.

Parallels can be found between this study and the work of others dating back as far as the 1950s which promotes the importance of the family doctors’ role in building personal relationships and understanding the individual [33–35]. These ideas about patient centredness began to redefine the role of the family doctor to recognize the mind and body as inextricably linked with the patient’s individuality at its core.

*“to restore the primacy of the person, one needs a medicine that puts the person in all his wholeness in the center of the stage and does not separate the disease from the man, and the man from his environment.”* ([35], p., 910]).

The findings of this study, particularly that the meaning of depression for older people and the way they communicate about it is built around their life contexts, echo these ideas and suggest they could be revisited by primary care health professionals in consultations with older people.

For McWhinney (1975) this is manifest in achieving a friendship-like relationship with personal knowledge [36], knowing the person both in good and ill health, treating the person before the illness so that the doctor gains understanding of the patients’ perceptions of their condition and what it means to them [37]. Evidence in the field often features the perspectives of clinicians and practice staff [11, 13, 14, 37, 38] whereas the focus of this study is solely the patient and their perspectives and may serve to enhance communication between patients’ and physicians so that their personalized life story is recognised. McWhinney proposes that achieving understanding on a personal level between patient and physician can “lead to quicker and more accurate diagnoses and more effective treatment” ([35], p., 910]) even though doctors have less time to listen and patients have higher expectations and make more demands of their physicians [37]. Evidence here which exposes how older patients both make sense of their depression and create meaning may be of value to GPs and other health professionals who spend more time with older patients.

It has been noted that recognising depression and articulating it can be challenging for older people who instead may describe a change in their sense of self in the context of their life stories [10]. Similarly, the life stories of participants in this study were integral to their constructions of depression, where their accounts would give insight into their experience of having depression. Identification of depression in clinical guidance is based on questionnaire scoring systems and identification of symptoms and may rely on the patient’s willingness to verbalise these and the extent to which they can articulate their narratives to GPs [39, 40]; this may not be suitable for all patients especially if they struggle to communicate about their depression in a clinical setting. This gold standard approach does not take into account the way older people conceptualise, accept and articulate depression which are the three main factors shown in this study to influence the way they communicate their depression to GPs. Opportunity could be found here for debate or reflection on the one size fits all approach taken in the guidance, which by the very nature of standardization is unavoidable.

This tension between standardisation and personalisation also points to a need for an approach which fits with older people’s ways of communicating about depression rather than expectations for them to report clinical symptoms. Confronting this may be daunting for GPs who may have little time to explore patients’ narratives in depth [41]. Likewise, their perspectives on managing older people with depression can be characterised by negativity when they have a lack of confidence in their expertise and tend to focus on problems and barriers [13, 37, 38]. They may also view depression as a consequence of patients’ life circumstances for which they cannot offer change and for which the treatments they can offer are limited in their effectiveness [42, 43]. The low response rate of 3 practices out of 169 agreeing to take part in the study could be a reflection of these factors. Development of approaches outside the GP consultation that fit with older people’s ways of communicating are in their infancy [9] but a lack of available services for older people means a time saving method of doing this that considers the demands of working in general practice could potentially support GPs.

The impact of stigma and assumptions about others’ views of depression may also act as a barrier to dialogue between older people and GPs [9, 10, 19]. Feeling a responsibility to avoid burdening other people has been recognised in those with depression [10, 19] where not taking responsibility to look after oneself is perceived by people with depression as a personality flaw or a reason to look down upon others with depression. Reluctance to “be a burden” (P13) prevented older people in this study from sharing their depression with family, friends and GPs and instead internalising it. During interviews the Striving to Understand category were responsive to probing and challenging whilst constructing their ideas, suggesting this stage of depression may be an opportunity to challenge these barriers. With suicide among older people estimated to be the tenth most common cause of death in the older population worldwide by 2020 [44, 45] the need to confront this perception is timely.

### Strengths and limitations

The typology is based on participants’ accounts of past episodes of depression or coming out of a recent episode so were likely to be recounting views with hindsight. Some described their depression as severe or mild but in the end this did not shape categories in the typology. While not being generalisable the study is potentially transferrable to other similar contexts and settings.

GPs recruited patients at their discretion and it was not known if a formal diagnosis of depression had been made. This method of recruitment was feasible within the research ethics framework as opposed to recruiting participants without a known diagnosis. Participants talked about their condition using a range of vocabulary. We recognise that older people who have not been given a diagnosis may talk about their condition and experiences differently to those who have, as they have not been given a label for their condition. Exploration of the impact of a diagnosis or no diagnosis on ways older people talked about depression was therefore not possible within the study design.

Data collection and analysis was undertaken by the lead author from a non-clinical perspective. This may have been valuable for patients who find it difficult to express their feelings to GPs and for addressing problems between older people and GPs relating to a condition that has become progressively more medicalised among older people who may see it as a non-medical problem.

The approach here is an unusual attempt to explore patients’ views with minimal influence or bias from the clinical setting or context, using methods that allow patients to lead the development of ideas in the data. Reporting older people’s perspectives in isolation underlines the importance of their voice being heard and allows an in-depth account of ways they communicate about depression.

### Implications for practice and research

This study raises the question, what help do older people need for their depression? The findings indicate that older people need flexible support depending on how they conceptualise depression and the extent to which they can articulate their problems and needs. Implications here are for a personalised approach to listening and decoding older people’s narratives about depression that recognises the importance of their situational and life contexts.

Future work is needed to develop strategies for GPs to quickly identify appropriate help for older people with depression that better fits with how they frame and talk about it and which also recognises the demands of general practice. Observations of GP consultations to consolidate the typology groups and further exploratory work to confirm acceptable support for each typology category is required. A model showing appropriate support for different categories of older people in the typology may have implications for other clinical and non-clinical practitioners, or others older people talk to, who may be able to listen for patterns in older people’s depression narratives and offer support, advise or signpost older people to getting the help they need.

## Conclusions

This study provides insight into how older people’s constructions of depression manifest in their depression narratives to increase understanding of ways they communicate about depression. It highlights the importance of recognising differences between older people in their understanding, acceptance and willingness to share their stories of depression and suggests the value of these differences as cues to determine appropriate support. The typology presented in this study may help GPs recognise patterns in patients’ narratives, their different conceptual positions regarding depression and explore their life contexts to gain more insight into their depression. This personalised approach may assist GPs when they need to try new communication strategies with patients or try to get to the core of what depression means to an individual to provide the best care for them.

## Additional file

Additional file 1: Initial interview topic guide for older people. Topic guide used with older people during in-depth interviews. (DOCX 16 kb)

## Abbreviations

AC: Professor Ann Crosland
CH: Dr.Catherine Hayes
GP: General Practitioner
IG: Dr. Isabel Gordon
JL: Professor Jonathan Ling
LR: Professor Louise Robinson
NHS: National Health Service
PHQ9: Patient Health Questionnaire PHQ9 for depression
