# Supplementary material for: Talking about depression during interactions with GPs: a qualitative study exploring older people’s accounts of their depression narratives
Source: BMC Fam Pract. 2018 Nov 3;19:173. doi: 10.1186/s12875-018-0857-8 (PMC6215358; doi:10.1186/s12875-018-0857-8)
Supplement: Supplementary file 1 — Initial interview topic guide for older people. Topic guide used with older people during in-depth interviews. (DOCX 16 kb) [file 12875_2018_857_MOESM1_ESM.docx]

**Initial interview topic guide for older people**

- Overall views of depression

(prompts: experiences of depression/ what depression is/ whether views have changed and if so why; influences over views of depression; are their views different during depressive episodes)

- Contextual influences over views of depression

(prompts: experiences of having depression in different contexts e.g. home, work, primary care, location, different social situations; views on how depression is diagnosed, how decisions are made about help needed for depression; experiences of historical and cultural attitudes depression)

- Experiences of visiting GP with depression

(prompts: experiences of seeing GP with depression -what has been good /bad/what could be improved; their role in decision making; whether experiences seeing GP have influenced the way they view depression)

- Talking to others about depression

(prompts: conversations with GPs– what has been good/bad; how depression was first brought up/ language used; decisions about how they talk to GP about depression/reasons for these decisions; would this be different if feeling depressed)

- What works well and what could be changed about how depression is managed in primary care

(prompt: changes that could be made and reasons for these views; changes to their role in decision making with GP)
